# Impact of antigen avoidance test for fibrotic hypersensitivity pneumonitis in stable phase

**DOI:** 10.1186/s13223-022-00748-1

**Published:** 2022-12-09

**Authors:** Ryo Okuda, Tamiko Takemura, Tae Iwasawa, Shota Kaburaki, Tomohisa Baba, Eri Hagiwara, Takashi Ogura

**Affiliations:** 1 Departments of Respiratory Medicine, Kanagawa Cardiovascular and Respiratory Center, 6-16-1 Tomioka-Higashi, Kanazawa-Ku, Yokohama, Japan; 2 Departments of Pathology, Kanagawa Cardiovascular and Respiratory Center, 6-16-1 Tomioka-Higashi, Kanazawa-Ku, Yokohama, 236-0051 Japan; 3 Departments of Radiology, Kanagawa Cardiovascular and Respiratory Center, 6-16-1 Tomioka-Higashi, Kanazawa-Ku, Yokohama, 236-0051 Japan

**Keywords:** Acute exacerbation, Bird fancier's lung, Causative antigen, Chronic hypersensitivity pneumonitis, Home-related hypersensitivity pneumonitis

## Abstract

**Background:**

The antigen avoidance has been used in the diagnosis and treatment of hypersensitivity pneumonitis (HP); however, its usefulness in stable fibrotic HP is controversial.

**Objective:**

To investigate the usefulness of the antigen avoidance test in patients with fibrotic HP in stable phase.

**Methods:**

The antigen avoidance test was conducted during a 2-week hospitalization comparing clinical parameters at admission and before discharge. A retrospective review of patients who underwent surgical lung biopsy or transbronchial lung cryobiopsy, who were diagnosed with fibrotic HP by multi-disciplinary discussion, and whose disease progression was stable for more than two months before the antigen avoidance test was done.

**Results:**

Between 2016 and 2021, 40 patients met the criteria, and 17 (43%) patients had a positive antigen avoidance test. The patients with positive in the antigen avoidance test had significantly greater annual forced vital capacity (FVC) decline than those with negative before the test (− 6.5% vs. − 0.3%, *p* = 0.045). The patients with positive antigen avoidance test had less annual FVC decline than those with negative in the year following the test (0.8% vs. − 5.0%, *p* = 0.048). The differences in annual improvement were found for serum Krebs von den Lungen-6 between the positive and negative patients in the year following the test (− 27% vs. − 5%, *p* = 0.049). In multivariate Cox hazard regression analysis, a negative result of the antigen avoidance test was a risk factor for death or acute exacerbation of fibrotic HP (HR = 0.26 [95% CI: 0.07–0.90], *p* = 0.034).

**Conclusions:**

In fibrotic HP patients in stable phase, the antigen avoidance test under a 2-week hospitalization was valuable in predicting prognosis.

## Introduction

Hypersensitivity pneumonitis (HP) is an interstitial lung disease caused by type III and type IV immune mechanisms [1]. HP is divided into acute, subacute, and chronic phases based on clinical type [2, 3]. However, the 2020 American Thoracic Society (ATS)/Japanese Respiratory Society (JRS)/The Latin American Thoracic Association (ALTA) diagnosis of HP in adults proposed two classifications: non-fibrotic and fibrotic HP [4]. Chronic HP and fibrotic HP are difficult to differentiate from idiopathic pulmonary fibrosis (IPF) [5]. Pathological findings are useful in differentiating IPF from fibrotic HP. The histopathological findings of fibrotic HP include poorly formed non-necrotizing granulomas, airway-centered fibrosis, bridging fibrosis, and cellular bronchiolitis with lymphocyte infiltration [4, 6, 7]; however, histopathological examination, such as surgical lung biopsy or transbronchial lung cryobiopsy (TBLC), cannot be performed in many patients for various reasons. Even with well-designed interview, high-resolution CT (HRCT) findings, multi-disciplinary discussion (MDD) and serum IgG testing against specific antigens, a definitive diagnosis of HP is still difficult in patents who have not undergone histopathological examination [4].

In Japanese clinical practice, the antigen avoidance test has long been performed by hospitalization for two weeks. During the 2 weeks of hospitalization, the patients are not allowed to leave the hospital at all, and various tests are performed during the hospital stay. Most parameters are investigated at the admission and two weeks after admission. A retrospective study of antigen avoidance in a 14-day hospitalization for patients with chronic HP showed improvement in several clinical parameters [8].

The antigen avoidance test is one of the important methods for diagnosing HP, especially acute HP and non-fibrotic HP; however, its usefulness in diagnosing chronic HP and fibrotic HP is controversial [9, 10]. Patients with fibrotic HP, including acute exacerbation of fibrotic HP or acute on fibrotic HP due to a temporary exposure to large amounts of inciting antigens, showed significant changes in the antigen avoidance test [11]; however, the efficacy of the antigen avoidance test in the stable phase of fibrotic HP is unknown. In this study, the efficacy of antigen avoidance test in patients with stable fibrosis HP was investigated.

## Methods

### Study design and population

This was a single center, retrospective study. Patients who underwent surgical lung biopsy or TBLC, had histopathological findings of fibrotic HP, and were diagnosed with fibrotic HP by MDD underwent the antigen avoidance test in our hospital between January 2016 and December 2021. Antigen avoidance test was not performed in patients where the inciting antigen has already been identified from a clinical history-taking, IgG testing, and inhalation challenge testing. Only in patients where the inciting antigen could not be identified was an antigen avoidance test performed. Patients who were judged by an attending physician to have a worsening fibrotic HP based on their symptoms, respiratory function test, HRCT imaging, or interstitial pneumonia markers within two months prior to the antigen avoidance test and who met the definition of acute exacerbation at admission were excluded. The definition of acute exacerbation was in accordance with the 2016 International Working Group Report on diagnostic criteria for acute exacerbation of IPF [12]. The study protocol was approved by the ethics committee of our hospital (KCRC-22-0032). Because this was a retrospective study, the signature of patient consent was waived. Information about this research was made available to the research subjects, and they were guaranteed the opportunity to refuse to have the research conducted.

### Antigen avoidance test

The decision to conduct the antigen avoidance test was made by attending physicians. For the 14-day antigen avoidance test, all patients were admitted to our hospital and isolated from their homes and workplaces. During the isolation period, they were not allowed to go out and were not treated with any new medications for fibrotic HP, such as prednisolone, immunosuppressants or anti-fibrotic agents. The medications taken prior to admission were continued during hospitalization. An attending physician consulted with patients, and a decision was made to conduct HRCT imaging before hospital discharge.

The parameters based on previous reports for the positive judgment of the antigen avoidance test were set as follows [8]:

1. A decrease by ≥ 12% in serum Krebs von den Lungen-6 (KL-6)
2. A decrease in white blood cell counts or C-reactive protein levels by ≥ 2.5% or ≥ 0.05 mg/dL, respectively
3. Increase in forced vital capacity (FVC) by ≥ 2.5%
4. Improvement in ground glass opacities and reticular shadows on HRCT imaging.

The above parameters were measured on the last day of antigen avoidance test. KL-6 is a mucin like glycoprotein with high molecular weight that is predominantly expressed by damaged alveolar type II cells and a good biomarker for disease behavior of HP [13, 14]. The improvement in HRCT findings was judged by a chest radiologist (T.I.) specializing in interstitial lung diseases. Of the above 4, ≥ 2 of these were considered positive for the antigen avoidance test.

All patients were instructed by family members or roommates to clean their homes, especially air conditioners and bathrooms, and discard down products while the patient was hospitalized, regardless of the test results. The annual changes in clinical parameters before and after antigen avoidance test were investigated. The prognosis, development of acute exacerbations, and treatment were obtained from their medical records.

### Evaluation of disease progression

FVC, serum KL-6, white blood cell counts and C-reactive protein were evaluated for annual disease progression. To investigate annual changes in parameters, values closest to the point one year before and one year after the antigen avoidance test were selected. Age, serum KL-6, FVC and pathological findings, such as fibroblastic foci and organizing pneumonia, were selected as risk factors for the development of death or acute exacerbation, with reference to previous reports [9, 15–17].

### Inciting antigens

Assessment of inciting antigens was performed in accordance with CHEST Guideline and Expert Panel Report [9]. The likelihood of the inciting antigens was divided into three categories: identifiable, indeterminate, and unidentified.

### Statistical analysis

For the comparison of continuous variables between the two groups, unpaired *t*-test or two-way analysis of covariance was used. As covariates, age, sex, and baseline parameters were mainly selected. The 2 × 2 contingency tables were used with Fisher's exact test. Cox hazard regression analysis was used to compare the time to event, parameters which were *p* < 0.2 in univariate Cox hazard regression analysis were selected for multivariate Cox hazard regression analysis, and the median baseline values were used as the cutoff. BellCurve for Excel was used for all statistics (Social Survey Research Information Co., Ltd.).

## Results

**Fig. 1:**
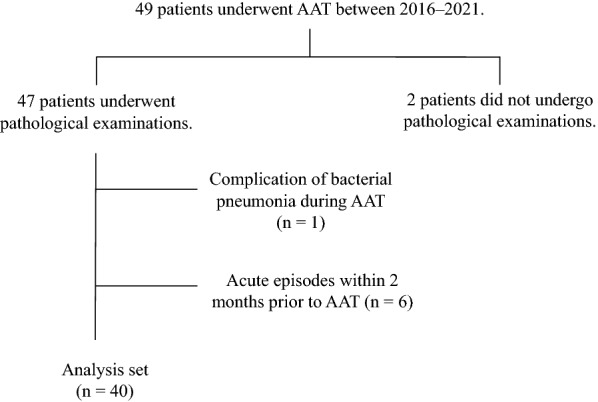
Flowchart showing patient inclusion criteria. AAT: antigen avoidance test

The antigen avoidance test was conducted in 49 consecutive patients with fibrotic HP. No patients refused to include this study. Two patients were excluded due to lack of histopathological survey. Because four patients had disease progression within two months before the antigen avoidance test and two patients had an acute exacerbation at admission, a total of six patients were excluded from this study because they were considered to have acute episodes. One patient also developed bacterial pneumonia during the antigen avoidance test and was excluded from this study. (Fig. 1). The median length of hospital stay was 13 days (Interquartile range: 10–14 days). The patient background was mid-70s in age, and patients had a mild fibrotic HP based on FVC and oxygen partial pressure in arterial blood. Assessment of the inciting antigen was identified in none, indeterminate in 14 patients, and unidentified in 26 patients. Of the indeterminate group, housing-associated fungi were suspected in 9 patients and avian antigens in 5 patients. Nine patients had fungi associated with their residence as their inciting antigen. HRCT imaging after the 2-week antigen avoidance test was performed in 34 patients (85%), and eight patients had improved ground glass opacities and reticular shadows. In the antigen avoidance test, the number of positive responses was 17 (43%). In the positive and negative groups, there were no significant differences in baseline age, sex, FVC, serum KL-6, and lymphocytes in bronchoalveolar lavage fluid (Table 1). Before the antigen avoidance test, three patients were treated with prednisolone, immunosuppressants, or anti-fibrotic agents, and none of them were on two drugs.

**Table 1:** Baseline characteristics

Subjects	Positive antigen avoidance test (n = 17)	Negative antigen avoidance test (n = 23)	*p-*value
Age (years)	73 (69–77)	74 (67–77)	0.94
Sex (M/F), n	7/ 10	13/ 9	0.34
Smoking history (Yes), n	5	13	0.12
Surgical lung biopsy, n	5	9	0.74
Cryobiopsy, n	13	17	1.00
CT performed after the antigen avoidance test, n	15	18	0.68
White blood cell (/μL)	6100 (5750–6910)	5630 (4990–7170)	0.71
C-reactive protein (mg/dL)	0.10 (0.06–0.15)	0.10 (0.05–0.28)	0.06
Serum Krebs von den Lungen-6 (U/mL)	1627 (821–2906)	1637 (1015–2509)	0.62
FVC (L)	1.92 (1.62–2.49)	2.12 (1.87–2.76)	0.26
FVC %pred (%)	80 (64–90)	78 (67–94)	0.91
PaO_2_ (Torr)	78.5 (68.9–85.6)	82.9 (75.0–91.8)	0.89
A-aDO_2_ (Torr)	19.0 (13.5–25.9)	18.0 (9.1–22.5)	0.19
BAL lymphocytes (%)	43 (24–64)	25 (17–45)	0.27
Anti-bird IgG antibodies (mgA/L)	14 (10–19)	12 (8–14)	0.13
Anti-Tricosporon asahii IgG antibody (CAI)	0.01 (0–0.04)	0.00 (0–0.05)	0.48
Airway-centered fibrosis, n	11	18	0.48
Lymphocyte-predominant bronchiolitis, n	11	15	1.00
Poorly formed granulomas, n	12	13	0.51
Fibroblastic foci, n	9	19	0.08
Organizing pneumonia, n	13	19	0.70
Prednisolone, n	0	1	
Immunosuppressant, n	0	1	
Anti-fibrotic agents, n	0	1	

Data are presented as median (IQR) or n (%). The reference ranges of anti-pigeon and anti-parrot IgG antibodies are 0–24.0 mgA/L and 0–14.0 mgA/L respectively

FVC: forced vital capacity; %pred: % predicted; PaO_2_: partial pressure of arterial oxygen; BAL: bronchoalveolar lavage; CAI: corrected absorbance index

The patients with a positive antigen avoidance test showed a significant improvement in serum KL-6 levels (− 6.5% vs. 1.7%) and white blood cell counts (− 12.6% vs. − 0.2%) compared to the negative group during the 2-week antigen avoidance test (Table 2). The average timings of annual survey were 9.0 months before the antigen avoidance test and 12.1 months after the test, respectively. In the year prior to the antigen avoidance test, patients with a positive antigen avoidance test had an annual decline in FVC (− 6.5% vs. − 0.3%, *p* = 0.045). In the year following the antigen avoidance test, the patients with a positive antigen avoidance test had an improvement in FVC (0.8% vs. − 5.0%, *p* = 0.048) and serum KL-6 levels (− 27% vs. − 5%, *p* = 0.049) compared to the negative group (Table 3). Out of 40 patients, 19 patients (48%) started or added a treatment within one year after the antigen avoidance test, and there was no significant difference in the frequency of new drug use according to the results of the antigen avoidance test. In the positive antigen avoidance test group, the additional or new drugs were prednisolone in 3 patients, tacrolimus in 3, and pirfenidone in 4; in the negative group, prednisolone was used in 10 patients, tacrolimus in 3, pirfenidone in 3, and nintedanib in 3. In addition, after the antigen avoidance test, the annual rate of FVC increased in the positive group with or without treatment, whereas it decreased in the negative group with or without treatment (Table 4). Using multivariate models, a negative result of the antigen avoidance test was a significant risk factor of death and acute exacerbation (Table 5).

**Table 2:** Changes during the 14-day antigen avoidance test

Parameters	Antigen avoidance test	
	Positive	Negative
delta FVC %pred (%)	2.4 ± 1.1 (n = 16)	0.4 ± 0.9 (n = 20)
delta Serum KL-6 (%)	− 6.5 ± 2.5 (n = 15)	1.7 ± 2.1 (n = 23)
delta White blood cell (%)	− 12.6 ± 2.9 (n = 17)	− 0.2 ± 3.3 (n = 23)
delta C-reactive protein (mg/dL)	0.02 ± 0.02 (n = 17)	− 0.08 ± 0.08 (n = 23)

Data are presented as mean ± standard error. Analysis of covariance was used with age, gender, and each baseline parameter as covariate

FVC: forced vital capacity; %pred: % predicted; KL-6: Krebs von den Lungen-6

**Table 3:** Annual changes in 1 year the antigen avoidance test

	Antigen avoidance test		*p*-value
	positive	Negative	
A. BEFORE			
delta FVC %pred (%)	− 6.5 ± 2.3 (n = 14)	− 0.3 ± 1.9 (n = 20)	0.045
delta Serum KL-6 (%)	32.5 ± 17.2 (n = 16)	13.6 ± 9.9 (n = 21)	0.32
delta White blood cell (%)	13.3 ± 4.6 (n = 16)	− 1.4 ± 6.6 (n = 21)	0.10
delta C-reactive protein (mg/dL)	− 0.08 ± 0.05 (n = 16)	0.00 ± 0.07 (n = 21)	0.66
B. AFTER			
delta FVC %pred (%)	0.8 ± 1.5 (n = 15)	− 5.0 ± 2.4 (n = 20)	0.048
delta Serum KL-6 (%)	− 27.2 ± 7.2 (n = 16)	− 5.2 ± 7.5 (n = 23)	0.049
delta White blood cell (%)	32.8 ± 10.6 (n = 16)	29.6 ± 8.8 (n = 23)	0.81
delta C-reactive protein (mg/dL)	0.00 ± 0.04 (n = 16)	0.08 ± 0.09 (n = 23)	0.44

Data are presented as mean ± standard error. The analysis of covariance was used with age, gender, and each baseline parameter as covariate

FVC: forced vital capacity; %pred: % predicted; KL-6: Krebs von den Lungen-6

**Table 4:** Annual FVC changes between the treatment and non-treatment groups in one year after the antigen avoidance test

Positive antigen avoidance test	n	delta FVC %pred (%)
With treatment	7	0.4 ± 2.3
Without treatment	8	1.1 ± 2.1

Negative antigen avoidance test	n	delta FVC %pred (%)
With treatment	11	− 3.0 ± 2.2
Without treatment	9	− 7.5 ± 4.7

Data are presented as mean ± standard error or n. The analysis of covariance was used with age, gender, and baseline FVC %pred as covariate

FVC: forced vital capacity; %pred: % predicted

**Table 5:** Cox hazard regression analysis for mortality or acute exacerbation in patients with fibrotic HP

Variables	Level	Univariate *p*	Multivariate		
			HR	95% CI	*p*
Age		0.169			
Smoking history	Never vs. Former and current	0.263			
Serum Krebs von den Lungen-6 (U/mL)	≥ 1630 vs. < 1630	0.678			
Forced vital capacity (%)	≥ 80 vs. < 80	0.137	0.46	0.153–1.41	0.174
Lymphocytes in BAL (%)	≥ 30 vs. < 30	0.218			
Airway-centered fibrosis		0.370			
Lymphocyte-predominant Bronchiolitis		0.615			
Poorly formed granulomas		0.338			
Fibroblastic foci		0.210			
Organizing pneumonia		0.109	6.49	0.75–56.0	0.089
Antigen avoidance test	Positive vs. Negative	0.081	0.26	0.07–0.90	0.034

HP: hypersensitivity pneumonitis; HR: hazard ration; CI: confidence interval; BAL: bronchoalveolar lavage

Since the patients were admitted to the hospital to isolate them from their living environments and only underwent blood sampling, respiratory function test, and HRCT imaging, no adverse events occurred. The patients became very bored during hospitalization. Three patients strongly requested to be discharged before their scheduled date, and after a consultation with the attending physician, one out of three patients was discharged earlier than planned.

## Discussion

The antigen avoidance test was conducted in patients with stable fibrotic HP having no identified inciting antigens. The positive group had a greater annual decline in FVC before the test, and the FVC and serum KL-6 increased in the year following the test compared to the negative group.

The ATS/JRS/ALAT diagnostic guidelines for HP published in 2020 did not include much information about the antigen avoidance test [4]. Antigen avoidance may improve clinical findings in patients with non-fibrotic HP; however, clinical improvements may not occur in patients with fibrotic HP [2, 3, 9]. In the clinical course of fibrotic HP, acute episodes and acute exacerbation occasionally occurred. Under acute conditions, significant changes may be observed in the antigen avoidance test. Therefore, to exclude patients with fibrotic HP in acute phase, patients with stable fibrotic HP for more than two months were included in this study. In accordance with previous reports, the thresholds of the antigen avoidance test used in this study were very small changes, such as a 2.5% decline in white blood cell counts and a 12% decline in serum KL-6 [8]. However, the positive and negative groups differed in annual FVC changes before and after the antigen avoidance test in this study.

Identifying the inciting antigen of fibrotic and non-fibrotic HP is also a difficult task. An inhalation challenge test is considered one of the methods to identify the inciting antigen, however, the inhalation challenge test is currently performed only in few specialized HP centers [18–20]. Specific IgG testing is also useful in identifying the inciting antigen; however, specific IgG testing does not directly identify antigens. Specific IgG testing can provide evidence of past and present exposure to antigens [4]. If symptoms and data improve with antigen avoidance, the antigen avoidance test provides a high confidence for the diagnosis of HP, and it also indicates that an inciting antigen exists in the patient's environment. The antigen avoidance test was effective for patients with non-fibrotic HP and acute HP because symptoms and data often improve rapidly by avoiding antigens [21]. Because a little change was observed in symptoms and data in patients with fibrotic HP, the usefulness of the antigen avoidance test for fibrotic HP was controversial [9]; however, in the current study, 17 (43%) patients with stable fibrotic HP were positive for the antigen avoidance test. Fibrotic HP can be diagnosed based on HRCT and pathological findings; however, it is not possible to identify the inciting antigen. It is also difficult to identify the inciting antigen in most patients with fibrotic HP by a clinical history-taking and IgG testing. About 40 patients of histopathology-proven fibrotic HP were diagnosed annually in our hospital. Out of 40, 15 patients underwent inhalation challenge test, and about eight patients underwent antigen avoidance test. The number of patients performing antigen avoidance testing is expected to increase based on results of this study.

Because the annual rate of FVC decline and prognosis favored fibrotic HP patients with a known inciting antigen over those with unknown inciting antigen, identifying the inciting antigen could be beneficial in predicting disease progression to fibrotic HP [21, 22]. In this study, although we have not been able to identify the inciting antigen, positive patients in the antigen avoidance test had reduced annual FVC decline after antigen avoidance test. Because patients with stable fibrotic HP and negative result in antigen avoidance test had greater decline in FVC and weaker improvement in serum KL-6 levels in the year following the antigen avoidance test, they possibly had a persistent antigen exposure, or their fibrotic lesions possibly progressed without antigen exposure.

For the prognosis and development of acute exacerbations, the results of the antigen avoidance test, not the background or histopathological findings, were risk factors. In the histopathological findings of chronic HP, the presence of fibroblastic foci (FF) was a poor prognostic factor [6, 15, 16]. However, in the study the presence of FF was not associated with prognosis. The percentage of patients who underwent surgical lung biopsy was low (35%), and most patients were diagnosed by TBLC; therefore, a difference in the method of histopathological specimens might have affected the results. Secondly, the small number of patients in this study could be one of the reasons. Finally, the results of the antigen avoidance test could have a greater prognostic impact than the presence of FF. The patients with a positive antigen avoidance test had a greater FVC decline of 6.5% in a year prior to the antigen avoidance test than those with a negative test, suggesting that they possibly had greater exposure to the inciting antigen before the test. Therefore, the antigen avoidance test seemed to be worthwhile in stable fibrotic HP patients with mild FVC decline.

Two weeks of isolation period is used as a standard in Japan due to the medical situation, and the isolation period in this study is also about 2 weeks [8]. A previous article reported a decline in interstitial pneumonia markers with an antigen avoidance period of one month [22]. Further studies are needed to determine the duration of antigen avoidance test. However, keeping the patients in the hospital for several weeks just for observation without any therapeutic intervention was associated with mental and physical distress. In addition, the number of patient beds is limited, and securing enough hospital beds for observation is difficult for any hospitals. For isolation, places aside from patient's homes and workplaces, alternative facilities, such as hotels, can also be considered. In the case of hotels, issues, such as the cost of stay and difficulty of checking the patient's condition, need to be resolved. The antigen avoidance test was simpler than other antigen identification tests, such as the inhalation challenge test, and the test risk was negligible, since patients were only hospitalized for isolation from their homes and workplaces.

In the present study, three patients were positive for anti-pigeon IgG antibody and none were positive for anti-Trichosporon asahii antibody. Although thresholds for anti-Trichosporon asahii antibody in patients with non-fibrotic HP have been reported, the threshold for anti-Trichosporon asahii antibody in patients with fibrotic HP is unknown. First, many species of fungi are known to cause fibrotic HP; however, antibodies against only one type of fungus have been measured. Second, no patients had birds as a pet at the time of the antigen avoidance and only 7% of patients were positive for pigeon antigen. Third, the cleaning of their home and workplaces might reduce the FVC decline. For these reasons, we speculated that the inciting antigen in many cases was more likely to be fungal than avian.

Our study had several limitations. First, this was a single center, retrospective study with a large bias in patient selection. To reduce the bias, patients who met the criteria were consecutively enrolled in this study, and prospective trials need to be conducted to determine the usefulness of the antigen avoidance test in the future. Second, the duration of hospitalization and the positive judgment criteria for the antigen avoidance test were not standardized. With regard to the duration of antigen avoidance, a hospital stay for more than 14 days was difficult under the medical conditions in Japan. As for the duration of hospitalization, reports from other countries are awaited; further studies on the positivity criteria are also needed. Finally, the treatment affected the clinical course after the antigen avoidance test. In the study, the analysis was stratified into the treated and untreated groups, and no differences in FVC changes were observed.

## Conclusions

The antigen avoidance test under a 2-week hospitalization was valuable in predicting prognosis, although changes in parameters resulting from the antigen avoidance test were very mild in fibrotic HP patients in stable phase.

## Data Availability

The datasets analyzed during the study are available from the corresponding author on reasonable request.

## Abbreviations

HP: Hypersensitivity pneumonitis
ATS: American Thoracic Society
JRS: Japanese Respiratory Society
ALTA: The Latin American Thoracic Association
IPF: Idiopathic pulmonary fibrosis
TBLC: Transbronchial lung cryobiopsy
HRCT: High-resolution CT
MDD: Multi-disciplinary discussion
FF: Fibroblastic foci
